# The Disinfection of Sick-Rooms

**Published:** 1917-09-01

**Authors:** 


### THE DISINFECTION OF SICK-ROOMS.

EVER and anon crops up afresh the question of the value of the methods commonly applied in the disinfection of rooms which have been occupied by patients suffering from infectious disease. The criticism of the modernist and the reformer is prone to be destructive, and it has become the fashion to decry these methods as being of doubtful utility. Recently the *Sanitary Record* has called attention to a discussion now taking place on the subject in American public-health papers. Dr. Adams, of Toronto, it is stated, has been giving his views but is unable to come to definite decisions; he is, of course, perfectly right in holding that it is of far greater importance to apply direct methods of disinfection during a patient's illness than to deal with his sick-room afterwards, but we cannot follow him in the contention that sick-room disinfection is "relatively of no importance." Still less can it be agreed that the practice of such disinfection has been continued, upon "sentimental" grounds, in order to quell the qualms of those who subsequently use the room, and that "it is this sentimental feeling that in the majority of instances will prevent many health authorities and officers from making up their minds to discontinue room disinfection." Whatever may be the views and feelings of the public, surely it cannot be allowed that public health authorities continue to practise, for the sake of peace and quietness, a mere fetichism worthy only of the medicine-man of a savage tribe.

Many causes have conduced to bring disinfection methods as often applied to rooms into disrepute and even contempt. First and foremost amongst them is the fact that, in view of modern knowledge of infection and immunity, the energies of preventive medicine have drifted into fresh scientific channels, and room-disinfection has become one only, and one of the less effective, of the means of preventing the spread of infection. There has been, too, a lack of adaptability in the light of knowledge; in some sanitary areas the same formal routine of wall-spraying or fumigation is carried out after a case of enteric fever as after one of diphtheria, whilst not infrequently in the room in which a fatal case of pulmonary tuberculosis has been nursed, nothing whatever is done. Less reliance being placed upon the value of these methods in the case of some infections, a slackness in their application has been allowed in all. Such slackness is soon noticed by the public: nothing can make a worse impression than the advent of an ignorant, self-opinionated labourer instructed, without supervision and whatever may be the circumstances, to carry out a perfunctory "disinfection" by the ignition of a sulphur candle. There is no excuse for allowing reliance to be placed upon a routine which is scientifically inapplicable, in order to save the expense of providing or using a disinfector or of the necessary destruction of infected articles.

The fact remains that, whilst we have learnt much, we have not yet learnt enough safely to dispense altogether with the need for sick-room disinfection. We know that it has its limitations; as the one and only effort on the part of a health authority worthy of the name it may fairly be called obsolete. We would prefer six hours' exposure of a wall-surface to bright sunlight to twelve hours' sulphur dioxide fumigation, or stripping and re-papering to a formalin spray; but means cannot always be chosen, and we must make use of the best available. The surfaces of walls and furniture, like clothing and bedding, can "hold" some forms of infection, and to argue to the contrary is absurd; to do so is to make a claim which cannot be upheld upon the knowledge we have of the life history of pathogenic organisms, and which is entirely opposed to the theories upon which sick-wards and surgical operating-rooms are constructed and used. Our homes are still too often far from ideal, and in our homes infectious diseases arise and have sometimes to be nursed. Fresh light is constantly being thrown upon the complex problems of infection, but as yet the disinfection of the sick-room must hold its place. When it is not needed it is dishonest and improper to allow it to be done perfunctorily or otherwise: when it is necessary it should be carried out intelligently and thoroughly by the full and careful use of the best means available.

